# Synergistic and Antagonistic Effects of Salinity and pH on Germination in Switchgrass (*Panicum virgatum* L.)

**DOI:** 10.1371/journal.pone.0085282

**Published:** 2014-01-14

**Authors:** Yuan Liu, Quanzhen Wang, Yunwei Zhang, Jian Cui, Guo Chen, Bao Xie, Chunhui Wu, Haitao Liu

**Affiliations:** 1 Department of Grassland Science, College of Animal Sci. and Techn., Northwest A&F University, Yangling, Shaanxi Province, P R China; 2 Institute of Grassland Science, College of Animal Science and Technology, China Agricultural University, Beijing, P R China; 3 Department of Plant Science, College of life Science, Northwest A&F University, Yangling, Shaanxi Province, P R China; 4 Lehrstuhl für Grünlandlehre, Technische Universität München, Am Hochanger 1, Freising-Weihenstephan, Germany; University of Louisville, United States of America

## Abstract

The effects of salt-alkaline mixed stress on switchgrass were investigated by evaluating seed germination and the proline, malondialdehyde (MDA) and soluble sugar contents in three switchgrass (*Panicum virgatum* L.) cultivars in order to identify which can be successfully produced on marginal lands affected by salt-alkaline mixed stress. The experimental conditions consisted of four levels of salinity (10, 60, 110 and 160 mM) and four pH levels (7.1, 8.3, 9.5 and 10.7). The effects of salt-alkaline mixed stress with equivalent coupling of the salinity and pH level on the switchgrass were explored via model analyses. Switchgrass was capable of germinating and surviving well in all treatments under low-alkaline pH (pH≤8.3), regardless of the salinity. However, seed germination and seedling growth were sharply reduced at higher pH values in conjunction with salinity. The salinity and pH had synergetic effects on the germination percentage, germination index, plumular length and the soluble sugar and proline contents in switchgrass. However, these two factors exhibited antagonistic effects on the radicular length of switchgrass. The combined effects of salinity and pH and the interactions between them should be considered when evaluating the strength of salt-alkaline mixed stress.

## Introduction

Salt-affected soils are present in a large portion of the marginal land, and millions of hectares of marginal cropland are limited by the high concentrations of salts in the soil [1], [2]. Currently, over half of the world's irrigated land and 20% of the cultivated land is affected by salinity [3]. Moreover, the world loses 0.25 to 0.5 Mha of agricultural land annually because of salt buildup, which mainly results from irrigation, especially in arid and semiarid areas [4]. Soil salinization and alkalization frequently occur together in nature, which is a major factor that limits crop production [5], [6]. Therefore, researching the effects of salt and alkaline stress on switchgrass, exploring the mechanisms of salt-alkaline tolerance and constructing suitable models of the effects of salt-alkaline stress on switchgrass are important goals.

Switchgrass (*Panicum virgatum* L.), an important species of tall prairie grass, is a warm-season C4 perennial rhizomatous grass that is native to North America [7], [8]. Historically, this grass has been classified into two main ecotypes based on its morphology and habitat, upland and lowland [9]. Switchgrass has an abundant lignocellulosic biomass that can be converted into liquid transportation fuels and other chemicals by fermentation [10], [11]. Switchgrass is considered a potential source of biomass feedstock for renewable energy production in marginal areas that would not compete with food production due to its broad adaptability, rapid growth rates, high yields, growth in less productive soils and high water use efficiency [12]–[15].

However, research on the salinity tolerance of switchgrass has just begun to address neutral salt stress, and alkaline stress and salt-alkaline mixed stress have thus far been neglected in these analyses. Despite their frequent concurrence, saline and alkaline stresses are distinct phenomena that can be induced separately in plants by exposure to specific inorganic ions. Previous reports have suggested that salt stress can be defined as the stress caused by neutral salts (NaCl and Na_2_SO_4_), and alkaline stress is the stress caused by alkaline salts (NaHCO_3_ and Na_2_CO_3_) [5], [16]. High levels of soil salinity and alkalinity interfere with both seed germination and plant growth [17], [18]. The weak osmotic gradient prevents water uptake during seed germination. Saline stress occurs at high ion concentrations irrespective of the pH, and it predominantly involves the neutral salts NaCl and Na_2_SO_4_. Its main effects are osmotic stress and ion overload [1], [19], [20], which can interfere with ion homeostasis in plants and disrupt the ionic balance [21], [22]. In contrast, alkaline stress occurs specifically at high pH levels and predominantly involves NaHCO_3_ and Na_2_CO_3_. Some reports have clearly demonstrated that alkaline salts are more destructive to plants than neutral salts, suggesting that a high pH environment also causes direct toxicity effects [5], [23]–[25]. The effects of saline and alkaline mixed stress mainly include salinity stress, pH stress and the interaction between them [5].

The successful establishment of plants largely depends on successful seed germination. The germination response of plants to environmental parameters determines the distribution of the plants in saline environments [26], and seed germination in a saline substrate is a legitimate criterion for selecting for tolerance in saline environments [27]. Moreover, high levels of alkalinity can be a limiting factor for seed germination [28], [29]. Plants can synthesize compatible organic solutes, proline and soluble sugar in the cytoplasm to survive and maintain their growth in saline conditions. This phenomenon is known as salinity tolerance [30]. Thus, most screening techniques that are used to assess this tolerance in plants use proline accumulation as one of the most important physiological parameters [31]. A large number of plant species have been shown to be capable of accumulating proline in response to salinity stress, and accumulation may play an important role in the defense against salinity stress [32]. In addition, the accumulation of other organic solutes, such as soluble sugars, has been observed during salinity stress in many plant species [33]. The content of MDA reflects the degree of damage to the plasma membrane. The membrane fat peroxidation function of plants can increase in adverse conditions, which would increase the content of MDA, which is one of the decomposition products. Thus, the MDA concentration has often been used as a marker to assess the severity of oxidative stress and the degree of plant sensitivity [34].

Three switchgrass cultivars were chosen in this study (Blackwell, Northern Oklahoma upland type, developed by Soil Conservation Service Plant Materials Center (SCS PMC), Manhattan, KS. Cave-in-rock, Lowland type from southern Illinois, developed by SCS PMC, Ellsberry, MO. Kanlow, Lowland type from central Oklahoma, developed by SCS PMC, Manhattan, KS [35]). In the present study, mixtures of neutral salts (NaCl and Na_2_SO_4_) and alkaline salts (NaHCO_3_ and Na_2_CO_3_) were used at various proportions to simulate a range of mixed salt and alkaline conditions. Sixteen types of mixed salt and alkaline conditions at different salinities and pH values were prepared to investigate the effects of mixed salt and alkaline stresses on switchgrass. The main objectives of the present study were (1) to evaluate the effects of saline and alkaline stress on switchgrass seed germination and seedling growth, (2) to test the influence of saline and alkaline stress on the soluble sugar, proline and MDA contents of switchgrass seedlings and (3) to determine how the integrative effects of salinity and pH coupling influence switchgrass seed germination and seedling growth.

## Results

### Salinity and pH are significantly correlated with the tested indices

The correlation coefficients of the germination percentage (GP), germination index (G_i_), plumular length (PL) and radicular length (RL), as well as the contents of soluble sugar (SS), MDA and proline are illustrated as functions of the salinity and pH (Table. 1). The salinity significantly negatively correlated with the germination percentage (r = −0.548), germination index (r = −0.638), plumular length (r = −0.622) and radicular length(r = −0.806), while it significantly positively correlated with the contents of soluble sugar (r = 0.271), MDA (r = 0.266) and proline (r = 0.744). The pH also significantly negatively correlated with the germination percentage (r = −0.354), germination index(r = −0.281), plumular length (r = −0.306) the contents of soluble sugar (r = −0.514) and proline(r = −0.288). It did not positively correlate with the radicular length and MDA. Increasing either the salinity or the pH reduced the germination percentage, germination index, plumular length and radicular length. Increased salinity resulted in increases in the soluble sugar, MDA and proline contents, while increases in the pH, i.e. alkaline conditions, reduced the soluble sugar and proline contents.

**Table 1:** Pearson correlation coefficients of the germination percentage, germination index and plumular length, radicular length and the contents of soluble sugar, MDA and proline with the salinity and pH.

	Germination percentage	Germination Index	Plumular length	Radicular length	Soluble sugar	MDA	Proline
Salinity concentration	−0.540***	−0.638***	−0.622***	−0.806***	0.271***	0.266**	0.744***
pH	−0.354***	−0.281***	−0.306***	−0.126	−0.514***	0.007	−0.288***
Germination percentage	1.000	0.965***	0.929***	0.774***	0.354***	−0.296***	−0.220**
Germination Index		1.000	0.907***	0.821***	0.175*	−0.280***	−0.305***
Plumular length			1.000	0.841***	0.332***	−0.291***	−0.258**
Radicular length				1.000	0.035	−0.360***	−0.378***
Soluble sugar					1.000	−0.009	0.318***
MDA						1.000	−0.019

N = 192, F-values are presented with significant differences: *P<0.05, **P<0.01, ***P<0.0001.

### Higher pH value in conjunction with salinity sharply reduced seed germination and seedling growth

**Figure 1:**
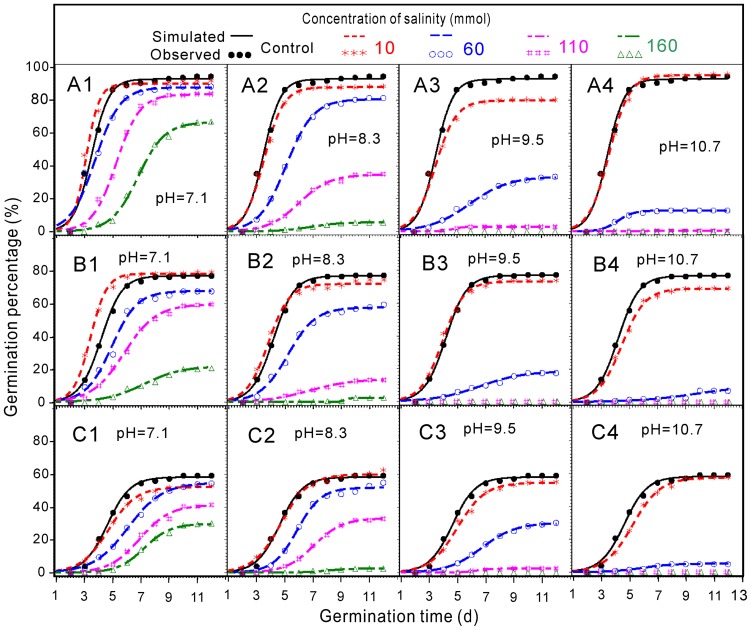
Simulated logistical curves showed the dynamic germination percentages under mixed salt-alkaline stress. A, B and C represent the cultivars, Blackwell, Cave-In-Rock and Kanlow switchgrass, respectively. All the logistic models were significant at *P*r<0.0001.

The switchgrass germination percentages (GP) at different salinity and pH levels are shown in Fig. 1. The significance of the curve fitting indicated that the experiments were accurate and reliable. The GP of the three switchgrass cultivars was highest in distilled water. The germination percentages were not markedly reduced at low salinity (≤10 mM), but they were significantly inhibited by high salinity and pH. When the salinity was higher than 60 mM, either the salinity or an increased pH sharply lowered the GP. At the same salinity concentration, the GP significantly decreased with increases in the pH. Furthermore, increasing the pH decreased the GP more than lowering the salinity. The three treatment groups designated as C4 (salinity = 110 mM, pH = 10.7), D3 (salinity = 160 mM, pH = 9.5) and D4 (salinity = 160 mM, pH = 10.7) did not show seed germination (Fig. 1). Additionally, the initial time to germination positively correlated with the salinity and pH. The GP of all three cultivars showed a similar dynamic tendency. The analysis of variance indicated that the seed germination percentage was significantly affected by the cultivar, saline concentration and pH for the individual effects, the pairwise effects and the interaction of the three variables (Table 2).

**Table 2:** Analyses of variance for the model of Germination percentage, Germination index, Plumular length and Radicular length, for each of the experimental factors and the interactions among them.

Source (factors)		Germination percentage (%)		Germination index		Plumular length (mm)		Radicular length (mm)	
	DF	*F* Value	*P*r>*F*	*F* Value	*P*r>*F*	*F* Value	*P*r>*F*	*F* Value	*P*r>*F*
C	2	169.81	<.0001	379.93	<.0001	15.35	<.0001	10.18	<.0001
S	5	803.06	<.0001	1310.8	<.0001	540.93	<.0001	1375.06	<.0001
P	5	491.27	<.0001	597.97	<.0001	260.74	<.0001	358.04	<.0001
C*S	10	13.60	<.0001	53.50	<.0001	8.74	<.0001	7.73	<.0001
S*P	7	26.17	<.0001	0.00	**1.0000**	20.08	<.0001	0.00	**1.0000**
C*P	10	18.67	<.0001	36.49	<.0001	2.96	0.0017	5.34	<.0001
C*S*P	14	3.77	<.0001	2.86	0.0004	1.37	**0.1648**	0.00	**1.0000**
Model	53	127.38	<.0001	186.85	<.0001	73.60	<.0001	141.06	<.0001

C, S and P represent the switchgrass cultivar, the saline concentration and the pH.

**Figure 2:**
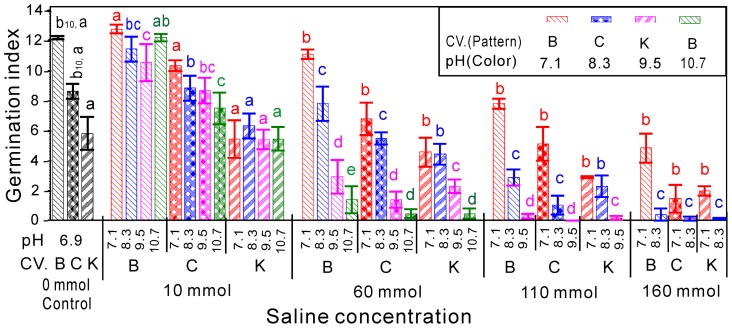
Effects of salinity and pH on the germination index. Each group of saline concentrations was separately compared with the control within the cultivar. The letters indicate differences at *P*<0.05 within the groups. B, C and K correspond to the capitalized the first letter of the switchgrass cultivars Blackwell, Cave-In-Rock and Kanlow.

The results of the switchgrass germination index (G_i_) at different salinity and pH levels are presented in Fig. 2. Like the GP, the G_i_ was not significantly altered by low salinity (≤10 mM), and it was even higher than the control when the pH = 7.1. However, the G_i_ decreased significantly as the salinity and pH increased, and the higher pH at a higher salinity level resulted in a more significant decrease in G_i_. Additionally, G_i_ did not change in the three treatment groups C4 (salinity = 110 mM, pH = 10.7), D3 (salinity = 160 mM, pH = 9.5) and D4 (salinity = 160 mM, pH = 10.7). The analysis of variance indicated that the cultivar, saline concentration and pH significantly affected the seed G_i_ individually, pairwise and as a combination of the three. However, the saline concentration and pH did not yield a pairwise effect (Table 2).

**Figure 3:**
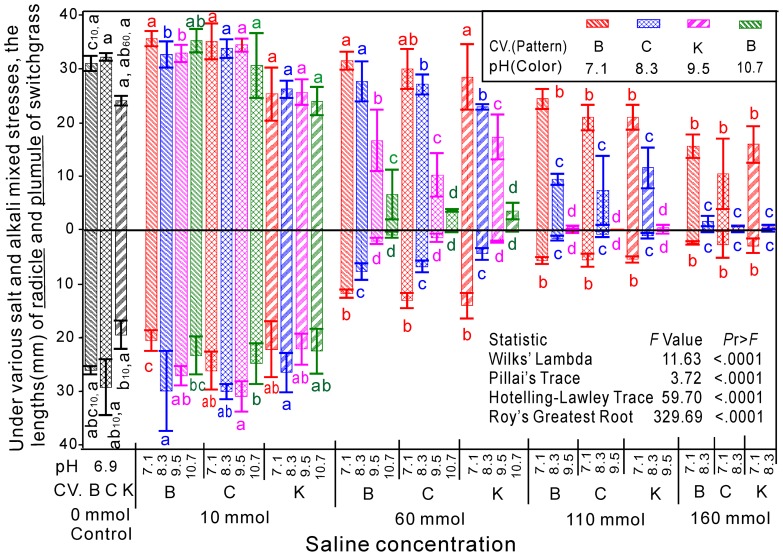
Effects of salinity and pH on the lengths of the plumules and radicles. Each saline concentration was separately compared with the control within the cultivar. The letters indicate the differences in the plumules or radicles within the groups. The letters indicate differences at *P*<0.05 within the groups. B, C and K correspond to the capitalized first letter of the switchgrass cultivars Blackwell, Cave-In-Rock and Kanlow.

The PL of the switchgrass negatively correlated with the salinity and pH, except when the salinity = 10 mM (Fig. 3). When salinity was 10 mM, the PL was longer than the control, particularly for the Blackwell cultivar, whose PL was significantly longer for all pH values. When the salinity≥60 mM, the PL decreased significantly as the pH increased. Similarly to the PL, the RL of switchgrass seedlings at low salt–alkaline mixed stresses (≤10 mM) did not significantly differ from the controls. However, the RL increased with the pH when the salinity = 10 mM, except at pH = 10.7. When the salinity exceeded 60 mM, either the salinity or the increasing pH sharply lowered the RL.

When the salinity≥60 mM, the growth of all radicles obviously differed from the control, but the plumule was still not significantly different when pH = 7.1(Fig. 3). Compared to the control, the radicle decreased more than the plumule. The analysis of variance indicated that the cultivar, saline concentration and pH significantly affected the PL and RL based on an analysis of the individual, pairwise and interaction of all three parameters. However, the saline concentration and pH did not yield a pairwise effect on the RL (Table 2).

### The soluble sugar, MDA and proline content were significantly affected by cultivar, saline concentration and pH

**Figure 4:**
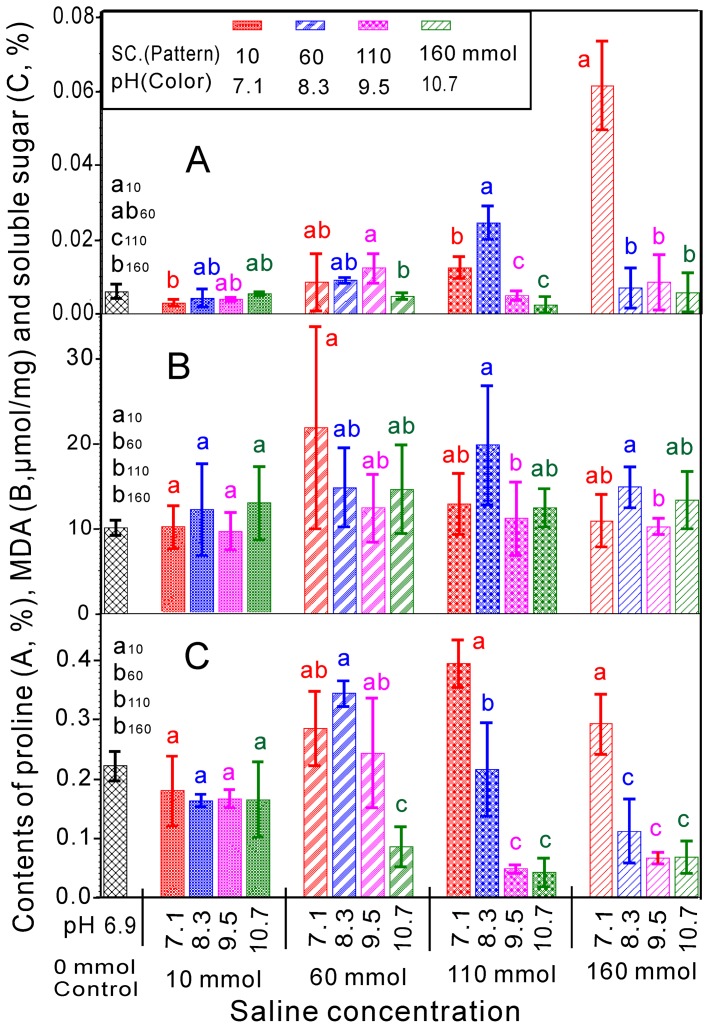
Contents of proline (A, %), MDA (B,μmol/mg) and soluble sugar (C, %) in switchgrass under salinity and alkali mixed stresses. The saline concentrations were separately compared with the control in each group. The letters indicate differences at *P*<0.05 within the groups.

The soluble sugar content (SS) in fresh switchgrass seedlings did not significantly increase at a lower salinity (≤10 mM), and the SS positively correlated with the salinity and pH (Fig. 4A). When the salinity = 60 mM, the SS significantly increased with the pH level, except at pH = 10.7. When the salinity = 110 mM, the SS increased until pH≥9.5. The highest SS occurred at salinity = 160 mM and pH = 7.1, which exceeded the control by more than 10-fold.

The average lipid peroxidation levels, as estimated from the MDA concentrations, were not significantly higher in response to salt-alkaline mixed stresses than in the control, except in groups B1 (salinity = 60, pH = 7.1), C2 (salinity = 110, pH = 8.3) and D2 (salinity = 160, pH = 8.3) (Fig. 4 B).

The proline content in the switchgrass seedlings was similar to that of the soluble sugar content. The proline content was not significantly increased at a low salinity (≤10 mM), and it positively correlated with the salinity and pH (Fig. 4C). When the salinity = 60 mM, the proline content increased with the pH level up to 8.3 and then decreased. When the salinity = 110 mM, the proline content was significantly higher than that of the control, and it negatively correlated with the pH level. The same pattern was observed when the salinity = 110 mM. The highest proline content occurred in group C1 (salinity = 110, pH = 7.1), which was only 2-fold higher than the control.

The analysis of variance indicated that the cultivar, saline concentration and pH significantly affected the soluble sugar, MDA and proline contents based on an analysis of the individual, pairwise and interaction of all three parameters. However, the saline concentration and pH did not show a pairwise effect on the MDA (Table 3). The multivariate analysis of variance indicated that the cultivar, saline concentration and pH significantly affect the measured indices (Table S1).

**Table 3:** Analyses of variance for the model of Suluble sugar, MDA and Prolne, for each of the experimental factors and the interactions among them.

Source (factors)		Soluble sugar		MDA		Proline	
	DF	*F* Value	*P*r>*F*	*F* Value	*P*r>*F*	*F* Value	*P*r>*F*
C	2	61.26	<.0001	261.26	<.0001	19.26	<.0001
S	5	104.10	<.0001	278.70	<.0001	740.85	<.0001
P	5	333.54	<.0001	256.69	<.0001	828.32	<.0001
C*S	10	8.04	<.0001	110.01	<.0001	32.29	<.0001
S*P	7	131.31	<.0001	0.00	**1.000**	1656.88	<.0001
C*P	10	6.47	<.0001	94.26	<.0001	46.98	<.0001
C*S*P	14	14.11	<.0001	83.22	<.0001	68.86	<.0001
Model	53	67.41	<.0001	120.09	<.0001	400.74	<.0001

C, S and P represent the switchgrass cultivar, the saline concentration and the pH, respectively.

### The synergetic and antagonistic effects in the physiological responses to equivalent coupling of salinity (X_1_) and pH (X_2_)

**Figure 5:**
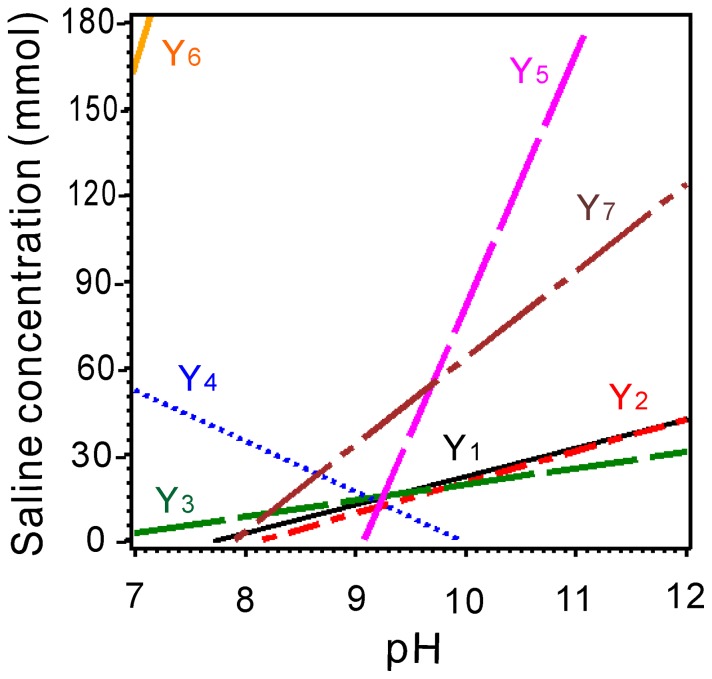
Ridgelines of the response surface models. Y_1_ to Y_7_ represent the germination percentage, germination index, plumular length and radicular length and the contents of soluble sugar, MDA and proline.

The germination percentage and germination index as well as the plumular and radicular length in switchgrass were considered as dependent variables, and the salinity and pH treatments were analyzed using two-variable quadratic regression models (Figure S1 and S2). The response surface and contour charts for the coupled effects of salinity and pH on the germination percentage show that the germination percentage was highest at low salinity and pH levels and then gradually decreased as the salinity and pH increased. Germination was not observed at high salinity and pH values (Figure S1 A). The contour chart shows that the salinity and pH had a stronger effect than the individual factors of salinity and pH with *k*>0 in the ridge line (Y_1_ in Fig. 5), which indicates that the salinity and pH had synergetic effects on the germination percentage of switchgrass (Figure S1 A). The germination index and plumular length showed a similar response to the coupled effects of salinity and pH (Figure S1 B and C), and the ridge lines also showed *k*>0 (Y_2_ and Y_3_ in Fig. 5). Therefore, the salinity and pH have synergetic effects on the germination index and plumular length of switchgrass (Figure S1).

The results shown in the surface and contour charts for the coupled effects of salinity and pH on the radicular length demonstrate that the radicular length was the longest at low salinity and pH levels (Figure S1 D). However, the radicular length positively correlated with the pH at low salinity. This effect differs from those observed for the germination percentage, germination index and plumular length. The red block in the response surface plots shows the results at higher salinity when the pH increased. In this case, the radicular length gradually decreased as the salinity and pH increased to ultimately prevent germination at high salinity and pH levels (Figure S1 D). Therefore, the contour chart shows that the salinity and pH have less of an effect individually than they do when combined, with *k*<0 for the ridge line (Y_4_ in Fig. 5). These data indicate that the salinity and pH have antagonistic effects on the radicular length of switchgrass.

The response surface and contour charts for the coupled effects of salinity and pH on the soluble sugar show that the soluble sugar content increased with the salinity at low pH. Simultaneously, the soluble sugar content increased first and then decreased as the pH increased (Figure S2 A). The soluble sugar content gradually decreased as the salinity and pH increased, until sugar content was no longer detected. Combined with *k*>0 in the ridge line (Y_5_ in Fig. 5), the salinity and pH had synergetic effects on the soluble sugar content of switchgrass. The MDA increased initially and then decreased as the salinity increased. Simultaneously, the MDA content decreased as the pH increased (Figure S2 B). At high salinity, the MDA content gradually decreased as the salinity and pH increased, which indicates that the salinity and pH have synergetic effects on the MDA content of switchgrass at high salinity combined with *k*>0 in the ridge line (Y_6_ in Fig. 5). The proline content positively correlated with salinity at a low pH and negatively correlated with salinity at a high pH. The proline content gradually decreased as the salinity and pH increased. Finally, proline and sugar was not detected at high salinity and pH values (Figure S2 C). The salinity and pH also had synergetic effects on the proline content of switchgrass combined with *k*>0 in the ridgeline (Y_7_ in Fig. 5). To show the effects more distinctly, the ridgelines of the response surface models were integrated and shown Fig. 5. The results show the synergetic and antagonistic effects due to the coupled effects of salinity and pH (Fig. 5).

## Discussion

The effects of mixed salts, especially mixed salt and alkali conditions, are more complex than those of a simple neutral salt or a simple alkali salt [5]. The effects of salinity and alkali mixed stresses mainly include salinity stress (S), pH stress (P) and the interaction between them (SP). However, the effects of the three stress factors on the physiological indices were different in magnitude. Research on the effects of mixed salt-alkali stress on the halophyte *Spartina alterniflora* (Poaceae) shows that S>P>SP [25], but research on the effects of mixed salt-alkali stress on *Aneurolepidium chinense* (Trin.) Kitag. shows that SP>S>P [5]. The results of this study indicated that S>P, which is consistent with the above points to some extent (Table 1, 4).

**Table 4:** Assignments of the treatments in the experimental design using a full combination design.

Saline concentration	10 (mmol)	60 (mmol)	110 (mmol)	160 (mmol)	Salt-alkaline mixed proportions NaCl: Na_2_SO_4_: NaHCO_3_: Na_2_CO_3_
A, pH = 7.1	1	5	9	13	1: 1: 0: 0
B, pH = 8.3	2	6	10	14	1: 2: 1: 0
C, pH = 9.5	3	7	11	15	1: 1: 1: 1
D, pH = 10.7	4	8	12	16	1: 1: 9: 9

The extra control was at 0 mmol salinity and pH 6.9. Four replicates were conducted for the 17 treatments. The last column indicated the salt-alkaline mixed proportions of NaCl: Na_2_SO_4_: NaHCO_3_: Na_2_CO_3_.

Salt stress is generally believed to inhibit plant growth by causing water deficiency and ion toxicity, among other factors [19], [36]. Heavy salt stress generally leads to a decrease in the germination percentage, growth arrest and even plant death [29], [37]. However, switchgrass maintains a relatively high germination percentage at a low-alkaline pH at any concentrations, confirming its salt tolerance and moderate alkali tolerance (Fig. 1). Similar to this study, Sautter [38] reported that Cave-in-Rock seeds germinated better under low salinity stress (≤85 mM NaCl), and germination was not observed at higher salt concentrations (≥171 mM NaCl).

High pH stress also appears to be a main factor in inhibiting germination and seedling growth [39], and this effect might be related to the effects of high pH on the transport of ABA [40]. In the present study, germination and seedling growth inhibition due to high pH(alkali)salt stress was stronger than the inhibition due to neutral salt stress at the same salinity, which is in agreement with previous studies [28], [29], [41], [42].

The deleterious effects of a high pH value or high salinity alone were significantly less than those of high pH in combination with high salinity [5]. This result suggested that the reciprocal enhancement between salt stress and alkali stress was a characteristic feature for salt-alkaline mixed stress, and this feature was most evidently reflected in the germination percentage. When the salinity was below 60 mM or the pH was 7.1, the relative germination percentage exceeded 80%. However, when the salinity was above 60 mM and the pH was above 8.3, the survival rates sharply declined as either the salinity or pH increased (Fig. 1).

The salt and alkali mixed stress show the effects of salinity and pH as illustrated by the inhibition of germination percentage, the germination index and the growth of the radicles and plumules in all three tested switchgrass cultivars. The seed germination of switchgrass was the highest in distilled water, and this group showed no indication of dormancy. Comparing the germination percentage of the three cultivars shows that B>C>K. The most effective inhibitory conditions on the germination potential were salinity = 180 mM and pH = 10.7. The germination percentage, germination index and growth of the radicles and plumules in all three cultivars negatively correlated with the salinity and pH. Seed germination was also strongly inhibited at the early stages of the experiment (Fig. 1, 2 and 3). The roots may be the primary target, as they are in direct contact with the mixed salt-alkaline treatment solution and were particularly sensitive to the treatment solution (Fig. 3).

In varying saline environments, the primary physiological response of plants consists of an osmotic adjustment via two processes: the accumulation of ions in the vacuole and the synthesis of compatible solutes in the cytosol [30]. Halophytes grown in saline conditions usually accumulate inorganic ions in the vacuoles to decrease the cell water potential because the energy consumption from absorbing inorganic ions is far less than that from synthesizing organic compounds [1]. Halophytes frequently accumulate large amounts of Na^+^ under salt-alkali stress [5], which would lead to Na^+^ toxicity in the cytosol and may limit the germination and seedling growth.

Halophytes can also synthesize compatible organic solutes, such as proline, solute sugar and polyalcohol, in the cytoplasm to prevent dehydration [30]. Furthermore, some of these organic solutes can also protect the biomacromolecules in the cytoplasm [1]. Our results indicate that the proline content and the soluble sugar content in fresh switchgrass seedlings positively correlated with the salinity and pH. This finding suggests that the induction of proline and soluble sugar synthesis is related not only to changes in the salinity but also to increases in the pH. Our results indicate that proline and soluble sugar act as osmolytes and protectants, and they may also have other roles related to alkali stress, which should be investigated further.

The membrane fat peroxidation function of plants can increase in adverse conditions. This process increases the content of MDA, which is one of the decomposition products [34]. The results presented here indicated that the mixed salt-alkali treatments induced incremental increases in the MDA content at both increasing salinity and pH conditions, except at a salinity of10 mM. Our results indicate that the membrane permeability of the seedlings was damaged by salinity and pH stress, which agrees with previous studies [2], [43]. The MDA accumulation as a result of increasing stress also indicated osmolyte induction in order to protect from damage. However, this induction apparently was not sufficient to totally block cell damage.

Some results show that high pH in combination with high salinity has more deleterious effects on the germination and seedling growth than a high pH value or high salinity alone [1], [29]. However, a clear model of the relationship between salinity and high pH has not yet been established. To analyze the effects of salt-alkali mixed stress on the germination and seedling growth of switchgrass, we used the two main factors, salinity and pH, to construct the models. The response surface and contour charts for the coupled effects of salinity and pH on the germination indices and physiological indices are shown in Figure S1 and S2. The ridgelines corresponding to the response surface for analyzing the synergetic and antagonistic effects are shown in Fig. 5.

The salinity and pH have synergetic effects on the germination percentage, germination index, plumular length as well as the contents of soluble sugar, MDA and proline of switchgrass. However, the salinity and pH have antagonistic effects on the radicular length of switchgrass. The ridgeline Y_1_ represents the germination percentage in response to the coupled effects of salinity and pH. Every point on the ridgeline Y_1_ shows that the salinity and pH have the same effects on the germination percentage, and the points higher than the line indicate that the salinity is the dominant factor. The points lower than the line indicate that the pH is the dominant factor. The ridgeline Y_1_ increases as the salinity and pH increase, which demonstrates that the salinity and pH have synergetic effects on the germination percentage. The ridgelines Y_1_, Y_2_ and Y_3_ indicate similar effects on the germination index and plumular length.

The results were combined for all three cultivars due to their similarity. Many different mechanisms have been suggested to explain the effect of the salinity and pH on seed germination and seedling growth. The main conclusion is that high pH stress can directly damage plant membrane construction, alter the availability of nutrients and disrupt the balance of ions and mineral nutrition [4], [25], [44]. In particular, the effects of high pH were clearly enhanced as the salinity increased (Fig. 1, 2, 3). The germination percentage, germination index and plumular length showed a significant negative correlation with the salinity and pH.

The ridgeline Y_4_ represents the radicular length in response to the coupled effects of salinity and pH. Interestingly, the ridgeline declines as the pH increases, which shows that the salinity and pH have antagonistic effects on the radicular length of switchgrass. Fig. 3 shows that the radicular length positively correlates with the pH when the salinity = 10 mM, which might be related to the effects of high pH on the transport of ABA [40]. The mechanism of the antagonistic effects of salinity and pH on the radicular length of switchgrass may be varied and deserves further investigation.

The ridgelines Y_5_, Y_6_ and Y_7_ represent the contents of soluble sugar, MDA and proline, respectively, in switchgrass in response to the coupled effects of salinity and pH. The salinity and pH showed synergetic effects on all of these indices, primarily due to the increase in salinity and pH. Halophytes synthesize compatible organic solutes in the cytoplasm, such as proline, carbohydrates and polyalcohol, to prevent dehydration. Additionally, the membrane fat peroxidation function in plants increases under adverse conditions, resulting in an increase in the MDA content. However, ridgeline Y_7_, which represents MDA, was very short. Simultaneously, the pairwise effects of salinity and pH on the MDA content were not significant. Therefore, the results for MDA were not highly reliable.

Objectively estimating the effects on plant growth for natural salt-alkalinized soil is difficult, especially for soil with a high pH. The models of salinity and pH may represent salt-alkali mixed stress. However, none of these indices could completely reflect the strength of salt-alkali mixed stress. Our results on the synthetic conditions combining salinity, pH and the interaction between them in salt-alkali soil may provide a new model to address this problem.

## Conclusions

In this study, switchgrass was found to be capable of germinating and surviving well under all treatments at low pH (pH≤8.3), regardless of the level of salinity. The germination and survival of this species is related to its ability to accumulate organic solutes (proline and soluble sugar). The deleterious effects of high pH or salinity alone were significantly less than the combined effects of high pH and salinity. We conclude that reciprocal enhancement between salinity and pH stress is a characteristic feature of salt-alkali mixed stress, which is demonstrated by the constructed models. The salinity and pH had synergetic effects on the germination percentage, germination index, plumular length and the contents of soluble sugar and proline, whereas the salinity and pH exhibited antagonistic effects on the radicular length of switchgrass. The synergetic and antagonistic effects of salinity and pH are new valuable models, which should be considered when evaluating the strengths of salt-alkali mixed stress.

## Materials and Methods

### Seed materials and Instrumentation

Three cultivars of switchgrass seeds, Blackwell, Cave-In-Rock and Kanlow, were used for this experiment and were donated by The Institute of Soil and Water Conservation of Chinese Academy and China Agricultural University. This study was carried out in the Grassland Science Department (Faculty of Animal Science & Technology, Northwest A&F University, Shaanxi Province, China).

The pH values of the mixed salt and alkaline solutions were adjusted using a pH meter (FE20, Mettler-Toledo AG, CH-8606 Greifensee, Switzerland). The Petri dishes and other glassware were oven dried by an electro-thermal constant-temperature oven (DHG-9140A, Shanghai Yiheng Instrument Co., Ltd., China). The seed germination and seedling growth were contained in a plant incubator (ZPW-400, Harbin DongTou SG-Tech Development Co., Ltd., China), and an electronic analytical balance (YP1200, Shanghai Science and Industrial Co., Ltd., China) was used to weigh the samples in this study.

### Design of the mixed salt and alkaline conditions

Two neutral salts (NaCl and Na_2_SO_4_) and two alkaline salts (NaHCO_3_ and Na_2_CO_3_) were selected based on the salt components of the extant salt-alkaline soil in the northern part of China [45], [46]. The four selected salts were mixed at various proportions according to the tolerability of switchgrass to the salt-alkaline stress and the varying ranges of salinity and pH in the soil. Four treatment groups (labeled A through D) were established with gradually increasing alkalinity (Table 4). All treatment groups had a 1∶1 molar ratio of monovalent salts (NaCl + NaHCO_3_) to divalent salts (Na_2_SO_4_ +Na_2_CO_3_); therefore, the total ion concentrations were the same throughout the treatments because the individual molar concentrations were the same. The groups A, B, C and D featured four levels of saline concentration, 10, 60, 110 and 160 mM, respectively. Using a full combination design and one control, seventeen salt-alkaline mixed stress treatments at varying combinations of salinity and pH were established. The three cultivars of switchgrass were submitted to the designed treatments.

### Seed Germination tests

The seed germination experiments were repeated four times with 50 seeds per treatment. The seeds were placed in 9-cm Petri dishes on two layers of Whatman No.1 filter paper (pH 7) moistened with 6 ml of distilled water or with the treatment solutions. The Petri dishes were encased with clear plastic wrap to prevent evaporation. The dishes were placed in a germinator (LRH-250-GS II, China) and subjected to an alternating diurnal regime of 12 h of light at 25°C and 12 h of dark at 20°C for 14 days. This temperature regime was chosen to represent the mid-spring temperatures, which corresponds to the time of year when this species germinates. The germinated seeds were counted every day for 12 days. The root length and shoot length were measured on the fourteenth day after sowing, and ten seedlings were chosen to measure from every Petri dish. If the total number of seedlings was less than ten, all seedlings were chosen for measurement. Seed germination was defined as the elongation of the coleoptile to 0.2 cm. To avoid changes in salinity, the weight of each Petri dish was recorded and distilled water was added daily to compensate for the lost weight.

The germination index was estimated according to the following formula:
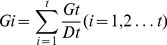
(1)where G*t* is the number of germinated seeds within the day, D*t* is the number of germination days and t is the total germination period (d).

### Physiological index measurements

All seedlings were harvested carefully after fourteen days of treatment and washed with distilled water. Any water remaining on the surface of the plants was blotted with filter paper. Each treatment contains three replicates. One hundred milligrams of fresh sample were used to measure the proline content according to the method described by Bates et al. [47]. Similarly, 100 mg of fresh sample were used to measure the contents of soluble sugar using anthrone [48], and 100 mg of fresh sample were used to measure the MDA content using the thiobarbituric acid (TBA) reaction, according to the method described by Madhava and Sresty [49].

### Statistical analysis

The germination percentages were simulated using the following logistic model [50]–[52]:
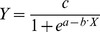
(2)where, *c*, *a* and *b* are constants.

The germination index, the plumular and radicular length and the contents of soluble sugar, MDA and proline were analyzed using the multivariate analysis of variance (MANOVA) test. Post hoc LSD tests were used to identify significantly different treatments.

The factors of salinity and pH were denoted by X_1_ and X_2_ in the analysis of synergetic and antagonistic effects. The dependent variables (Y), germination percentage, germination index, plumular length and radicular length as well as the contents of soluble sugar, MDA and proline were approached and analyzed via two-variable (X_1_ and X_2_) quadratic regression models as in [50]–[52]:
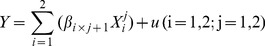
(3)where β is a constant. The response surface and contour charts were graphed (Fig. S. 1 and 2). For the equivalent coupled effects of X_1_ and X_2_, the following equation was used:



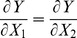
(4)Linear models of X_1_ with X_2_ were then obtained:
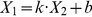
(5)where *b* is a constant. The models (5) present the ridgelines (Fig. 5) that correspond to the response surface models (3) for analyzing the synergetic and antagonistic effects. The analyses and graphical procedures specified above were all performed using SAS (v8.2) [53].

## Supporting Information

Figure S1 Response surface plots showing the synergetic and antagonistic effects of salinity and pH on germination percentage (A), germination index (B), plumular (C) and radicular length (D) in switchgrass.
(TIF)

Figure S2 Response surface plots showing the synergetic and antagonistic effects of salinity and pH on the content of sugar (A), MDA (B) and proline (C) in switchgrass.
(TIF)

Table S1 Multivariate Analysis of Variance – MANOVA Test Criteria and F Approximations for the Hypothesis of No Overall Effect. H =  Type III SSCP Matrix for cultivar, saline concentration and pH.
(DOC)
