# Supplementary figures and images for: Synergistic and Antagonistic Effects of Salinity and pH on Germination in Switchgrass (Panicum virgatum L.)
Source: PLoS One. 2014 Jan 14;9(1):e85282. doi: 10.1371/journal.pone.0085282 (PMC3891870; doi:10.1371/journal.pone.0085282)

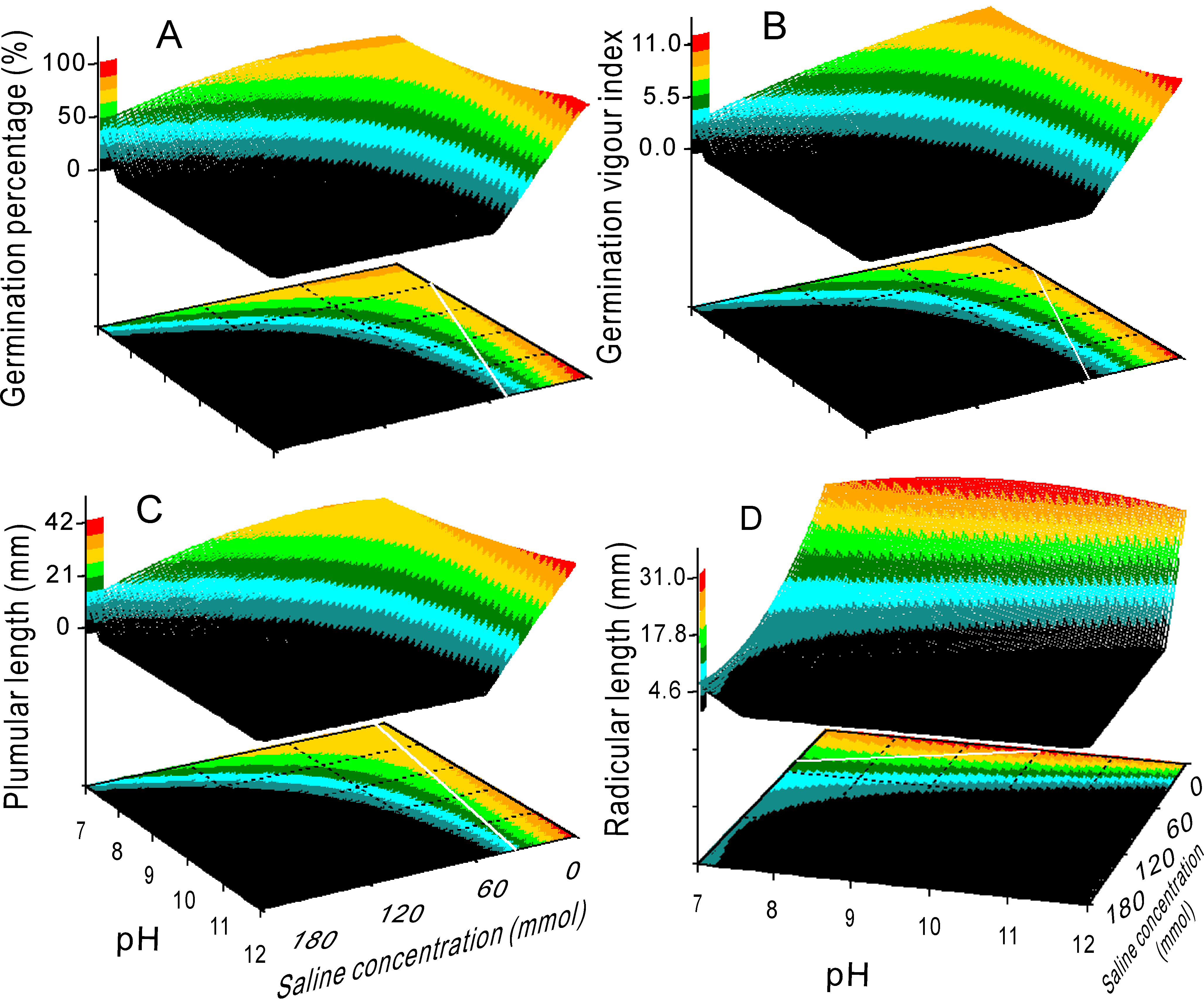

Supplement: Figure S1 — Response surface plots showing the synergetic and antagonistic effects of salinity and pH on germination percentage (A), germination index (B), plumular (C) and radicular length (D) in switchgrass. (TIF) [file pone.0085282.s001.tif]

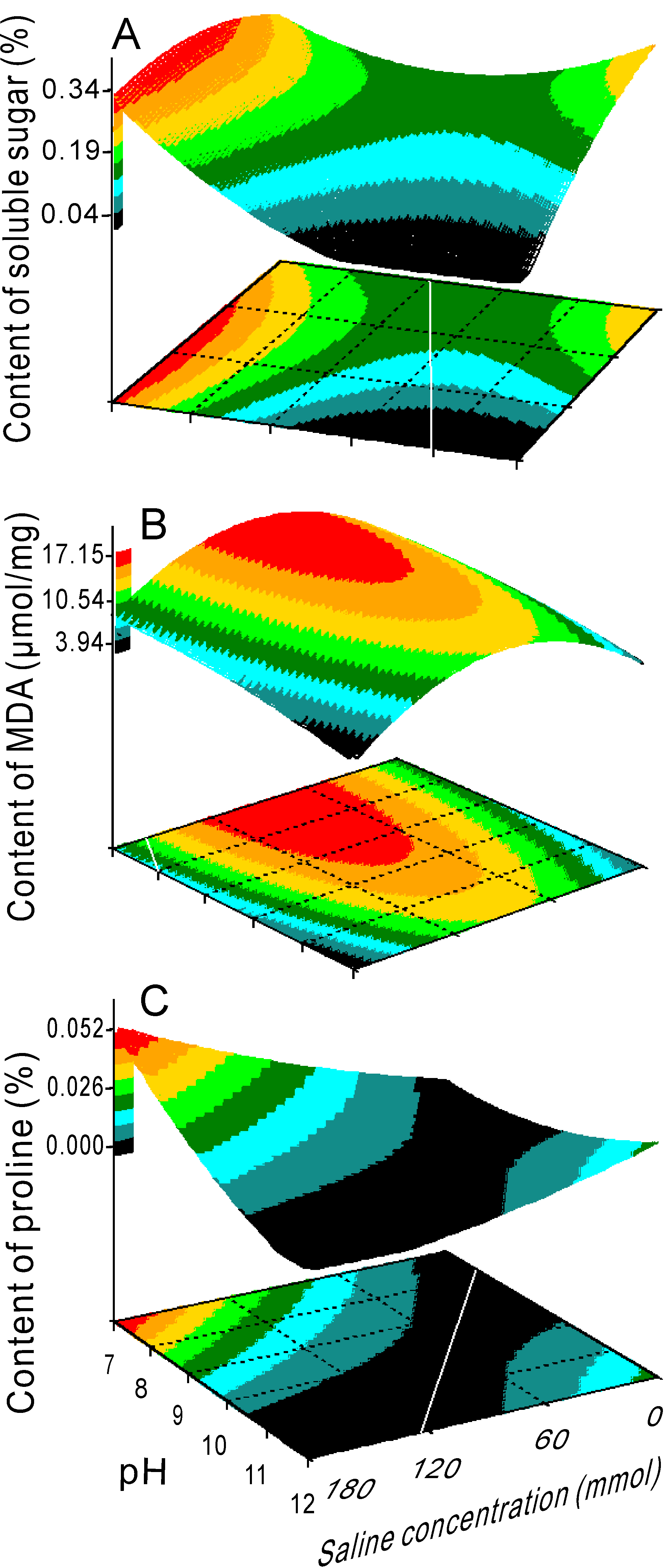

Supplement: Figure S2 — Response surface plots showing the synergetic and antagonistic effects of salinity and pH on the content of sugar (A), MDA (B) and proline (C) in switchgrass. (TIF) [file pone.0085282.s002.tif]
