# Supplementary material for: Synergistic and Antagonistic Effects of Salinity and pH on Germination in Switchgrass (Panicum virgatum L.)
Source: PLoS One. 2014 Jan 14;9(1):e85282. doi: 10.1371/journal.pone.0085282 (PMC3891870; doi:10.1371/journal.pone.0085282)
Supplement: Table S1 — Multivariate Analysis of Variance – MANOVA Test Criteria and F Approximations for the Hypothesis of No Overall Effect. H = Type III SSCP Matrix for cultivar, saline concentration and pH. (DOC) [file pone.0085282.s003.doc]

Table S1.

Multivariate Analysis of Variance - MANOVA Test Criteria and F Approximations for the Hypothesis of No Overall Effect. H = Type III SSCP Matrix for cultivar, saline concentration and pH. NOTE: F Statistic for Roy's Greatest Root is an upper bound. F Statistic for Wilks' Lambda is exact.

| Factors | Statistic | Wilks' Lambda | Pillai's Trace | Hotelling-Lawley Trace | Roy's Greatest Root |
| --- | --- | --- | --- | --- | --- |
|  | Num DF | 14 | 14 | 14 | 7 |
| Cultivar | *F* Value | 20.58 | 17.61 | 23.72 | 42.93 |
|  | *P*r > *F* | <.0001 | <.0001 | <.0001 | <.0001 |
| Saline concentration | *F* Value | 67.10 | 32.23 | 136.75 | 381.00 |
|  | *P*r > *F* | <.0001 | <.0001 | <.0001 | <.0001 |
| pH | *F* Value | 14.56 | 11.35 | 18.39 | 39.86 |
|  | *P*r > *F* | <.0001 | <.0001 | <.0001 | <.0001 |
